# Primary Esophageal Lymphoma: Clinical Experience in Diagnosis and Treatment

**DOI:** 10.7759/cureus.17628

**Published:** 2021-09-01

**Authors:** Junchi Qu, Yanyan Zhuang, Dandan Zheng, Fengting Huang, Shineng Zhang

**Affiliations:** 1 Department of Gastroenterology, Sun Yat-Sen Memorial Hospital, Sun Yat-Sen University, Guangzhou, CHN; 2 Guangdong Provincial Key Laboratory of Malignant Tumor Epigenetics and Gene Regulation, Sun Yat-Sen Memorial Hospital, Sun Yat-Sen University, Guangzhou, CHN; 3 Department of Gastroenterology, Sun Yat-sen Memorial Hospital, Sun Yat-sen University, Guangzhou, CHN

**Keywords:** primary esophageal lymphoma, prognosis, computed tomography, endoscopy, treatment

## Abstract

Primary esophageal lymphoma is a rare malignant tumor, which is often misdiagnosed. To improve the diagnosis and treatment of this disease, we presented one case admitted at our institution and a literature review of primary esophageal lymphoma cases, including the clinical data, features of imaging, endoscopy and pathology, treatment, and prognosis. The symptoms were non-specific. Under endoscopy, most of the lesions were located in the middle and lower segment of the esophagus, behaving as ulcers, polyps, or submucosal masses, always accompanying with esophageal stricture. The diagnosis of primary esophageal lymphoma was highly dependent on pathological and immunohistochemical examination, hence stacked sampling was suggested to improve the positive rate of mucosal biopsy. Combination of chemotherapy and radiotherapy may be the first choice of treatment, surgical and endoscopic resections may be an alternative solution as well. The therapeutic effect and prognosis were slightly better than those of other esophageal malignant tumors.

## Introduction

Primary esophageal lymphoma is a rare malignant tumor. Most of the primary malignant tumors in the esophagus are squamous cell carcinoma (about 98%), rarely adenocarcinoma, sarcoma, and lymphoma. The digestive tract is the most frequently involved extranodal organ, accounting for about 10%-20% of lymphoma, which includes stomach (48%-50%), small intestine (30%-37%), and esophagus (1%) [1]. It is reported that most primary esophageal lymphoma occurs in patients over 50 years old, mostly in women [2]. However, the clinical manifestations of esophageal lymphoma are variable. It is often misdiagnosed. Besides, the treatment approaches vary individually. However, an appropriate diagnosis and treatment strategy has not been established.

In this paper, the clinical data of 15 cases of primary esophageal lymphoma were collected and analyzed retrospectively, in which one case was admitted to our institution and 14 cases were reported in the literatures [2-14]. To improve the diagnosis and treatment of this disease, a systematic analysis was carried out, highlighting the clinical, endoscopic, and pathological features.

## Materials and methods

Patients and methods

**Figure 1:**
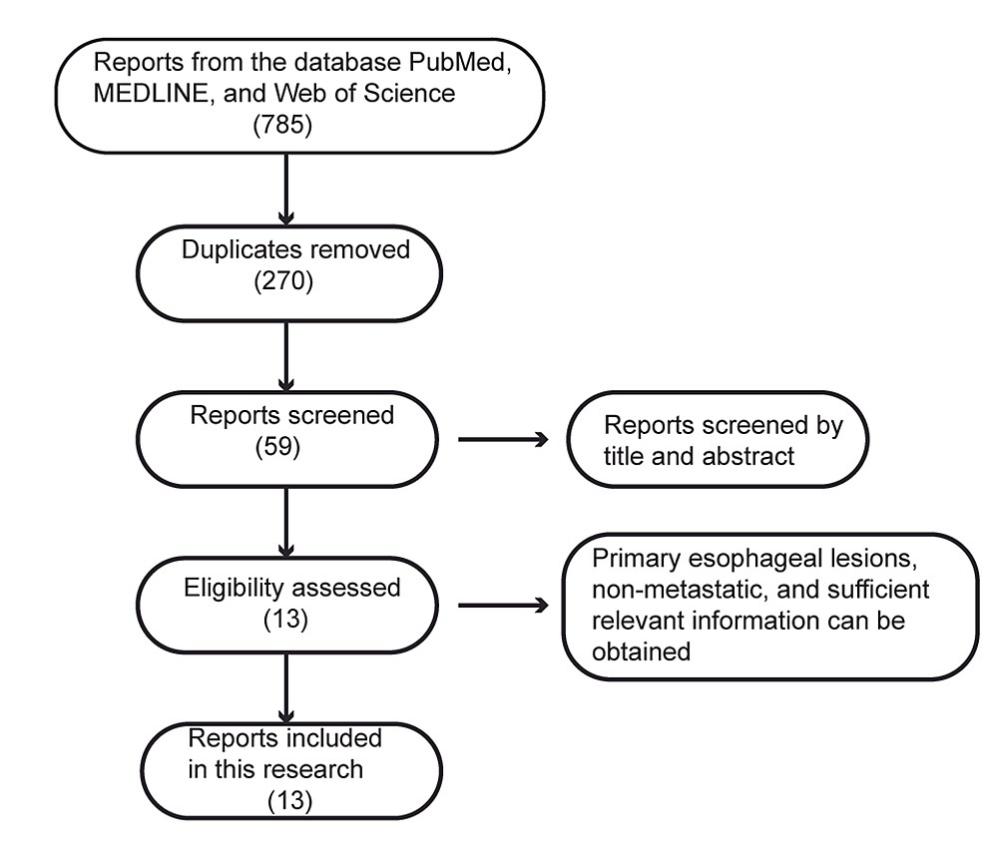
The flowchart of literatures screening An overview of the screening steps is presented as a flowchart.

We collected esophageal tumor specimens, imaging data, and endoscopic data of patients with primary esophageal lymphoma in Sun Yat-Sen Memorial Hospital, Sun Yat-Sen University from January 1980 to December 2020. Only one patient was found in our hospital. The specimens were obtained through endoscopic biopsies with the informed consent of the patient before chemotherapy with histological confirmation. In addition, we searched PubMed, MEDLINE, and Web of Science databases for related articles published between January 1961 and December 2020 with the key words of “primary esophageal lymphoma” or “primary esophagus lymphoma.” A total of 785 possible relevant literature reports were found and further selected according to the following inclusion and exclusion criteria after removing the duplicates. Inclusion criteria: 1. The lesion occurred in the esophagus. 2. The lesion was primary to the esophagus, not metastatic. 3. Histologically confirmed. Exclusion criteria: 1. Non-lymphomatic lesions. 2. Metastatic lesions to the esophagus. 3. Unable to obtain the necessary information for the case (such as endoscopy, CT, pathology, and immunohistochemical results). After being screened, 13 reports including a total of 14 patients with primary esophageal lymphoma were selected. The clinical characteristics of symptoms, endoscopic and imaging features were extracted from the articles (Figure 1)

Statistical analysis

Statistical analysis was carried out using SPSS version 25.0 software (SPSS, Inc., Chicago, IL, USA). Continuous variables were compared using analysis of variance, and were expressed as the mean ± standard deviation.

## Results

Patients clinical characteristics

The median age of 15 patients with primary esophageal lymphoma was 57 years old (25-77 years), including 13 males (86.7%) and two females (13.3%). Of the 15 patients, one patient was asymptomatic (6.7%) and was diagnosed during a routine health checkup. Dysphagia was the most common symptom (12 patients, 80.0%), while other symptoms such as pharyngalgia (three patients, 20.0%), hematochezia (three patients, 20.0%), nausea, and vomiting (two patients, 13.3%) could also be observed. There was one patient (6.7%) with tracheoesophageal fistula. Unexpectedly, none of the patients had acid reflux or heartburn, the common symptoms in other esophageal illnesses (Table 1). The levels of digestive tract tumor markers, including alpha-fetoprotein (AFP), carcinoembryonic antigen (CEA), and cancer antigen 19-9 (CA19-9), were within the normal range. The patient admitted to our hospital in November 2017 was a 25-year-old man suffering from dysphagia, nausea, vomiting, and pharyngalgia but denied acid reflux or heartburn (Table 1).

**Table 1:** Clinical features of the 15 patients with primary esophageal lymphoma *Patient No.1 was treated in our hospital. F: female; M: male; N: no; Y: yes

Patient No.	Age	Gender	Clinical features							Ref. No.
			Dysphagia	Acid reflux or heartburn	Pharyngalgia	Hematochezia	Nausea and vomiting	Tracheoesophageal fistula	Weight loss	
1*	25	F	Y	N	Y	N	Y	N	N	-
2	41	M	Y	N	N	N	N	N	N	2
3	77	M	Y	N	N	N	N	N	N	2
4	29	M	Y	N	N	N	N	N	N	3
5	60	M	Y	N	N	N	N	N	N	4
6	52	M	Y	N	N	N	N	N	N	5
7	59	M	N	N	N	Y	N	N	N	6
8	57	M	Y	N	N	N	N	N	N	7
9	69	F	N	N	N	N	N	N	N	8
10	60	M	Y	N	N	N	N	Y	Y	9
11	75	M	Y	N	N	N	Y	N	N	10
12	40	M	Y	N	Y	N	N	N	Y	11
13	64	M	Y	N	N	Y	N	N	Y	12
14	49	M	N	N	N	Y	N	N	N	13
15	39	M	Y	N	Y	N	N	N	N	14

Image features

**Figure 2:**
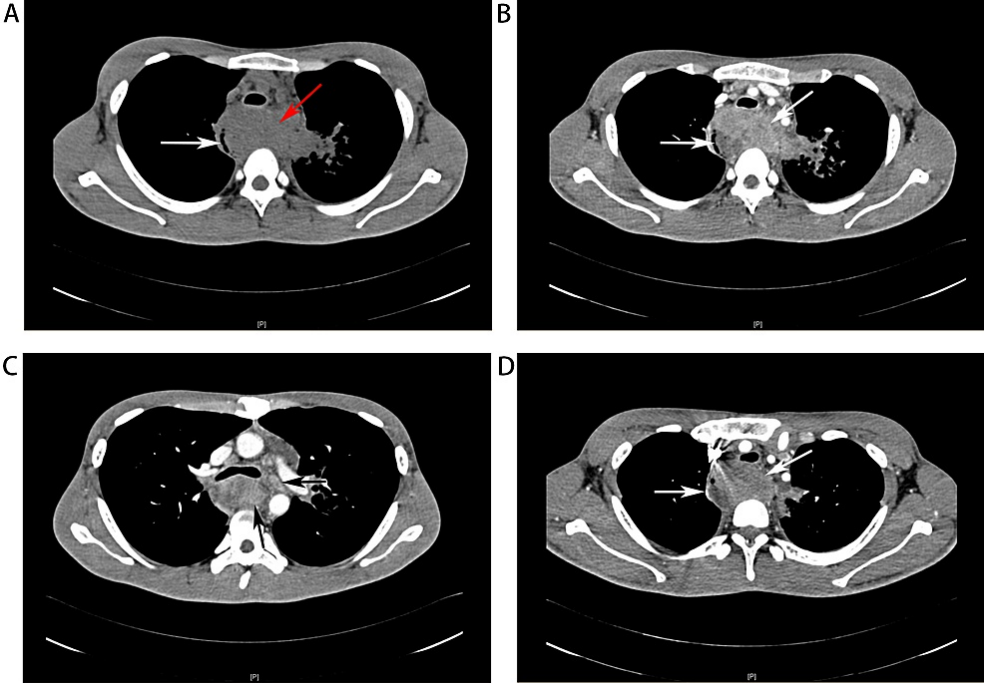
Imaging characteristics of primary esophageal lymphoma (A) CT revealing that the lesion was a low-density mass with obvious thickening of esophageal wall (red arrow) and stenosis of esophageal lumen (white arrow). (B) Contrast-enhanced CT revealing a mild heterogeneous enhanced lesion during the venous phase (white arrow). (C) Contrast-enhanced CT showing a mediastinal lymph node enlargement (black arrow), fusion with esophageal lesions, unclear boundary during the venous phase. (D) Contrast-enhanced CT showing a heterogeneous enhanced lesion during the arterial phase (white arrow).

All patients underwent CT scan. Eight of them underwent additional enhanced CT. Generally, the CT manifestations of 15 patients with primary esophageal lymphoma were similar, while the endoscopic features were various. The tumors were all represented as low-density lesions on a plain CT scan, except two cases that were not described. Enhanced CT scan showed moderate and above enhancement of the lesions in five patients (62.5%) and mild enhancement in three patients (37.5%), among the eight patients who underwent enhanced CT scan. We also reported the enlargement of lymph nodes in the esophageal drainage area. A total of seven patients (46.7%) had local lymph node enlargement (all of them had enlarged mediastinal lymph nodes and two patients had hilar lymph node enlargement simultaneously). Interestingly, one patient had distant lymph nodes enlargement such as aortocaval lymph nodes and both common iliac lymph nodes without local lymph node enlargement (Table 2). The contrast-enhanced CT of the patient admitted to our institution showed an unclear boundary and low-density mass with mild heterogeneous enhancement in the middle part of the esophagus, around which enlarged and merged mediastinal lymph nodes could be observed, leading to esophageal stenosis (Figure 2).

**Table 2:** Imaging and endoscopic features of the 15 patients with primary esophageal lymphoma ND: not described; N: no; Y: yes; P: proximal; M: middle; D: distal

Patient No.	CT				Gastroscopy				
	Density	Enhancement	Infiltration depth/cm	Lymphadenectasis	Site of tumor	Gross appearance	Mucosal erosion	Contact bleeding	Esophageal stricture
1	Low	Mild heterogeneous enhancement	47	Mediastinal and hilar lymph nodes	M	Protruding and	Y	Y	Y
						Ulcerating			
2	Low	Moderate-enhanced	ND	N	M	Ulcerating	Y	ND	Y
3	Low	ND	ND	Subcarinal lymph nodes	M	Multiple polyps	N	ND	N
4	Low	ND	4	Paraaortic lymph node	D	Protruding and circumferential ulcerating	Y	ND	Y
5	Low	ND	ND	Pretracheal, precarinal, and bilateral hilar lymph nodes	M-D	Circumferential	ND	ND	Y
						Ulcerating			
6	Low	Poorly enhanced	ND	N	P	Polyps	Y	ND	Y
7	Low	Weakly contrast-enhanced	15	Paraesophageal lymph nodes	P-D	Huge	N	N	N
						Submucosal mass			
8	Low	ND	2	N	M-D	Infiltrating tumor	N	N	N
9	Low	ND	2	N	M	Submucosal	N	N	N
10	Low	Enhanced	ND	Mediastinal lymph nodes	M	Fungating and ulcerating	Y	Y	N
11	Low	Mild contrast-enhanced	15.5	N	M-D	Lobulated submucosal mass	N	N	N
12	Low	Enhanced	ND	N	M-D	Polyps and longitudinal ulcerating	Y	ND	Y

Endoscopy manifestations

**Figure 3:**
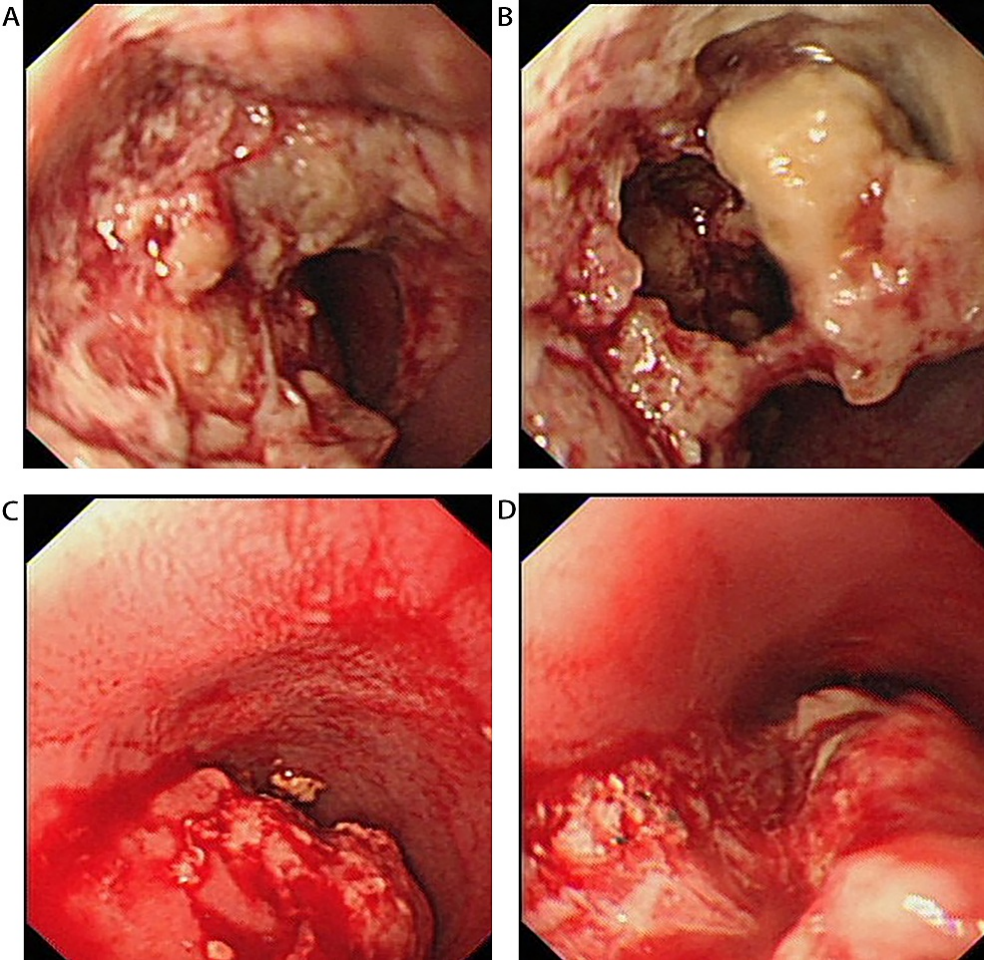
Endoscopic features of primary esophageal lymphoma (A) (B) Gastroscopy shows infiltrating masses in the middle part of the esophagus with erosion, ulcer, and significant lumen stenosis. (C) (D) Gastroscopy shows that the mass of esophagus is brittle and has contact bleeding.

Endoscopic manifestations varied in 15 patients, including ulcerating (53.3%), polyps (26.7%), submucosal mass (26.7%), protruding (20.0%), fungating (6.7%), and infiltrating (6.7%). Most occurred in the middle and lower esophagus (11 patients, 73.3%), while in two patients (13.3%), occurred in the upper esophagus. In particular, one patient (7.1%) occurred in the upper and middle esophagus and another patient manifested as a huge submucosal mass that involved the whole segment of the esophagus. Furthermore, mucosal congestion and erosion were found in seven patients (46.7%), esophageal stricture in seven patients (46.7%), and contact bleeding in two patients (13.3%) (Table 2). The case treated in our hospital showed an infiltrative mass with obvious congestion and erosion in the mucosa, accompanying with obvious esophageal stricture (Figure 3).

Treatments and follow-up

**Figure 4:**
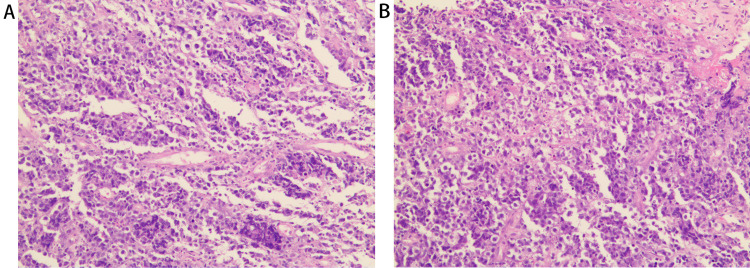
Pathological characteristics of primary esophageal lymphoma (A) (B) Histopathology showed that lymphoid tumor cells were diffusely distributed, with large cells, rich cytoplasm, partially transparent, large nuclei, obvious atypia, and easy mitosis. All representative images shown are stained with hematoxylin and eosin; magnification: ×200.

Three patients underwent resection of the tumors. One patient underwent surgical procedure for the removal of the esophageal mass due to the misdiagnosis of leiomyoma by endoscopy. Two patients underwent endoscopic submucosal dissection (ESD), which manifested as oval or fusiform submucosal masses with clear boundary and soft texture. The other 12 patients underwent endoscopic forceps biopsy. The histopathology of 15 patients was B cell phenotype in 10 cases (66.7%) and T cell phenotype in five cases (33.3%). B cell phenotype included diffuse large B cell lymphoma (DLBL) (33.3%), mucosa-associated lymphoid tissue lymphoma (MALT) (33.3%), and small B cell lymphoma (6.7%), while T cell phenotype included natural killer (NK)/T cell lymphoma (13.3%), anaplastic large cell lymphoma (ALCL) (13.3%), and peripheral large T cell lymphoma (6.7%). Both phenotypes had lymphocyte-like cells infiltrate (Table 3). Histopathologically, diffuse distribution of lymphoid tumor cells with rich cytoplasm, large nucleus, and obvious atypia was microscopically found in the patient admitted in our hospital. The immunohistochemical features were CD30 (+), CD43 (+), leukocyte common antigen (LCA) (+), T-cell intracellular antigen-1 (TIA-1) (+), anaplastic lymphoma kinase (ALK) (+), Epstein-Barr virus-encoded RNAs (EBER) (-), which was in accordance with the characteristics of ALK-positive anaplastic large cell lymphoma (Figure 4).

**Table 3:** Pathological characteristics and treatment of the 15 patients with primary esophageal lymphoma IHC: immunohistochemistry; ALCL: anaplastic large cell lymphoma; DLBL: diffuse large B-cell lymphoma; MALT: mucosa-associated lymphoid tissue lymphoma; R: radiotherapy; C: chemotherapy; S: surgery; ESD: endoscopic submucosal dissection; ND: not described; LCA: leukocyte common antigen; TIA: T-cell intracellular antigen-1; ALK: anaplastic lymphoma kinase; EBERs: Epstein-Barr virus-encoded RNA

Patient No.	Gross appearance	Histopathology	IHC (positive)	FISH	Treatment	Follow-up
1	Protruding and ulcerating	ALCL	CD30, CD43, LCA, TIA-1, ALK	EBER(-)	C	2 years, no recurrence
2	Ulcerating	DLBL	LCA, CD20, CD79	ND	S+C+R	16 months, no recurrence
3	Multiple polyps	DLBL	ND	ND	Untreated	ND
4	Protruding and circumferential ulcerating	ALCL	CD30, CD3, ALK, MUM1	t (2;5) translocation	C	13 months, complete remission
5	Circumferential ulcerating	DLBL	CD20, MIB‑1/Ki67 98%	ND	R+C	3 years, disease free
6	Polyps	NK/T cell	CD3, CD56, Ki67 90%, TIA‑1	ND	C	24 months, asymptomatic
7	Huge submucosal mass	MALT	CD20, BCL-2	MALT1：3/API2：2=16%	R	6 months, histological negativity
8	Infiltrating tumor	Small B cell	CD20, CD10, CD79, BCL-2	ND	C	1 year, symptom relief
9	Submucosal	MALT	CD20, BCL-2	ND	ESD	57 months, no recurrence
10	Fungating and ulcerating	DLBL	CD20, CD10, CD45, CD79a, BCL-2	ND	C	6 months, partial remission
11	Lobulated submucosal mass	MALT	CD20, CD21, CD23, PAX-5, BCL-2	EBER(-)	S	8 months, asymptomatic and no recurrence
12	Polyps and longitudinal ulceration	NK/T cell	CD2, CD3, CD43, CD8, CD56, TIA-1	EBER(+)	R+C	1 month, died of cerebral hemorrhage
13	Polyps and ulcerating	Peripheral large T cell	T cells with loss of CD5 expression	ND	C	10 months, disease free
14	Subepithelial	MALT	CD20, BCL-2, BCL-6, Ki67 5%	ND	C	6 months, no recurrence
15	Protruding and ulcerating	DLBL	CD79a, Mib-1	ND	C	3 years, complete remission

**Figure 5:**
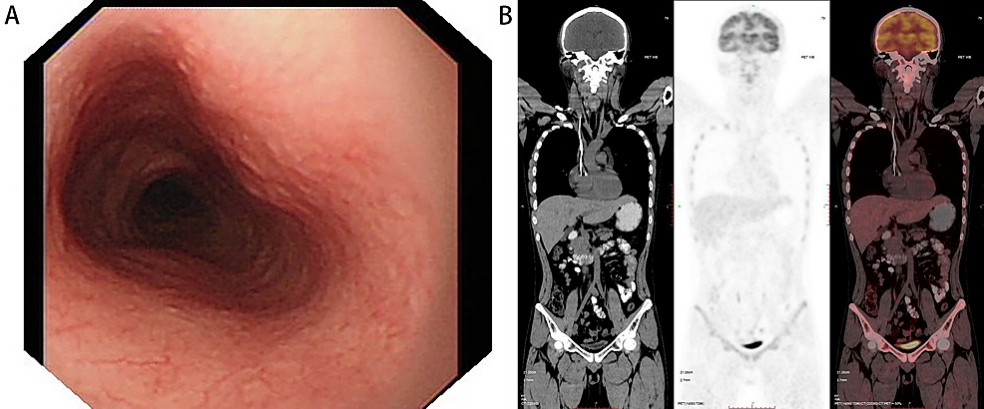
Follow-up of primary esophageal lymphoma after eight courses of CHOP regimen chemotherapy (A) Gastroscopy showed that the original esophageal tumor disappeared completely, the mucous membrane of each segment of the esophagus was smooth, there was no ulcer and esophageal stricture. (B) PET-CT showed that there was no obvious abnormality in glucose metabolism in all segments of esophagus, no metastatic hypermetabolic foci were found in paraesophageal lymph nodes, mediastinal lymph nodes, and other parts of the body, and no tumor recurrence and metastasis were found. PET-CT: positron emission tomography-CT; CHOP: cytophosphane, hydroxydaunorubicin, oncovin, and prednisone

In addition to one patient who refused treatment and follow-up, all the patients received individualized therapy. Eight patients received chemotherapy alone and two patients received radiotherapy combined with chemotherapy. One patient received radiotherapy alone. One patient underwent ESD and one patient underwent conventional surgery. Meanwhile, one patient received surgery combined with chemotherapy and radiotherapy. During the follow-up from half a year to one year, except for one patient who died of cerebral hemorrhage during treatment, there was no recurrence. The patient in our institution had a good prognosis after receiving eight courses of CHOP (Cyclophosphamide, Hydroxydaunorubicin-also known as doxorubicin, Oncovin-also known as vincristine, and Prednisone) chemotherapy. The primary tumor disappeared completely after therapy under endoscopy. Positron emission tomography-CT showed that there was no obvious abnormality in the whole esophagus and other parts of the body (Figure 5).

## Discussion

Primary esophageal lymphoma is very rare and a few cases have been reported worldwide. Dawson et al. concluded that the diagnostic criteria of primary esophageal lymphoma were as follows: 1. mainly involved esophagus; 2. only local lymph nodes were involved; no peripheral and mediastinal lymph nodes were involved; 3. normal liver and spleen; 4. normal granulocyte count [15]. However, 46.7% (7/15) of the patients in the study had enlarged mediastinal lymph nodes, histologically proved to be primary esophageal lymphoma. It seems that negative mediastinal lymph nodes are not necessary for the diagnosis of esophageal lymph nodes, which still needs to be further studied.

There are many difficulties in the clinical diagnosis of esophageal lymphoma. On the one hand, the clinical symptoms, endoscopic manifestations, and imaging features of esophageal lymphoma are variable, non-specific, and lack of specific markers, which often contribute to misdiagnosis. According to our study, primary esophageal lymphoma mainly manifests protruding mass with or without ulcer under endoscopy and dysphagia but no reflux or heartburn in clinical symptoms. And enhanced CT scan mainly shows uneven or relatively uniform mild to moderate enhancement. Patients with similar clinical manifestations as above may suggest the possibility of primary esophageal lymphoma. On the other hand, it is difficult to obtain ideal biopsy specimens because the main part of primary esophageal lymphoma often locates in the submucosa. The accurate diagnosis rates oaf endoscopy, percutaneous ultrasound, CT, and gastrointestinal barium meal were only 88.1%, 52%, 67%, and 83%, respectively [16]. With the low positive biopsy rate of the disease, clinicians should always take into account this disease when they encounter suspected patients. And taking stacked forceps biopsy which means repeated biopsies from the same lesion may help to avoid misdiagnosis [13].

There is no commonly acknowledged standard treatment for primary esophageal lymphoma. Chemotherapy, radiotherapy, surgery, and endoscopic resection are optional [17,18]. Recently, chemotherapy and radiotherapy are favorable and have become the first-line therapy, especially the CHOP regimen. There are different therapeutic options for different types of primary esophageal lymphoma. For instance, extranodal NK/T cell lymphoma is a highly invasive tumor with rapid progress and poor prognosis, a combination of radiotherapy and chemotherapy is of priority. And treatment scheme containing asparaginase is better than the conventional treatment schemes such as CHOP. With a total remission rate of 88.2% and a complete remission (CR) rate of 52.9%, Zhou et al. used DDGP (gemcitabine, pegaspargase, cisplatin, and dexamethasone) regimen to treat 17 patients with recurrent and refractory extranodal NK/T cell lymphoma [19]. Besides, the addition of rituximab to CHOP (R-CHOP) has proven to have higher response rates and improve the clinical outcome of patient with DLBL and R-CHOP [20]. Lee reported that cyclophosphamide, vincristine, and prednisolone (CVP) chemotherapy played a positive impact on MALT treatment [13]. Besides, MALT lymphoma is also sensitive to radiotherapy and 20-40 Gy is effective for tumor response and patient survival [21]. Once complications as obstruction, bleeding, or perforation occur, patients may need surgical intervention. Endoscopic esophageal dilatation or stent is alternative to treat stenosis [3]. Blystad et al. considered that bone marrow transplantation can be performed in the event of recurrence of esophageal lymphoma [22].

In summary, we put forth some new point of views of treatment and diagnosis in primary esophageal lymphoma. Partly consistent with Dawson, we propose the diagnostic criteria as follows: 1. The lesion mainly locates in the esophagus; 2. Only local and nearby lymph nodes (including mediastinal lymph nodes ) are involved; no peripheral and distant ones are involved; 3. Without hepatosplenomegaly; 4. Mild to moderate enhancement of lesion under CT scan; 5. Normal tumor markers; 6. Pathologically confirmed. For the good response, chemotherapy alone is recommended for patients with B cell phenotype. For aggressiveness, combination therapy may be a better choice for patients with T cell phenotype. Surgery and ESD are suitable options for patients with a local lesion, absence of lymph nodes enlargement. However, because of the limited cases of primary esophageal lymphoma, these recommendations have limitations. Further validation of these findings may be necessary for future research.

## Conclusions

The manifestations of primary esophageal lymphoma were non-specific. Thus, the diagnosis was highly dependent on pathological and immunohistochemical examination. Stacked sampling may be necessary to improve the positive rate. Combination of chemotherapy and radiotherapy may be the first choice of treatment, while surgical and endoscopic resections are also alternative solutions. The therapeutic effect and prognosis were slightly better than those of other esophageal malignant tumors.
